# Genetic Diversity Study on Geographical Populations of the Multipurpose Species *Elsholtzia stauntonii* Using Transferable Microsatellite Markers

**DOI:** 10.3389/fpls.2022.903674

**Published:** 2022-05-12

**Authors:** Chenxing Zhang, Chunfeng Jia, Xinru Liu, Hanqing Zhao, Lu Hou, Meng Li, Binbin Cui, Yingyue Li

**Affiliations:** ^1^National Engineering Research Center of Tree Breeding and Ecological Restoration, College of Biological Sciences and Technology, Beijing Forestry University, Beijing, China; ^2^Key Laboratory of Genetics and Breeding in Forest Trees and Ornamental Plants, Ministry of Education, College of Biological Sciences and Technology, Beijing Forestry University, Beijing, China; ^3^The Tree and Ornamental Plant Breeding and Biotechnology Laboratory of National Forestry and Grassland Administration, Beijing Forestry University, Beijing, China; ^4^College of Biochemistry and Environmental Engineering, Baoding University, Baoding, China

**Keywords:** cross-transferability, *Elsholtzia stauntonii* Benth., simple sequence repeat (SSR) markers, genetic diversity, population structure

## Abstract

*Elsholtzia stauntonii* Benth. (Lamiaceae) is an economically important ornamental, medicinal and aromatic plant species. To meet the increasing market demand for *E. stauntonii*, it is necessary to assess genetic diversity within the species to accelerate the process of genetic improvement. Analysis of the transferability of simple sequence repeat (SSR) markers from related species or genera is a fast and economical method to evaluate diversity, and can ensure the availability of molecular markers in crops with limited genomic resources. In this study, the cross-genera transferability of 497 SSR markers selected from other members of the Lamiaceae (*Salvia* L., *Perilla* L., *Mentha* L., *Hyptis* Jacq., *Leonurus* L., *Pogostemon* Desf., *Rosmarinus* L., and *Scutella* L.) to *E. stauntonii* was 9.05% (45 primers). Among the 45 transferable markers, 10 markers revealed relatively high polymorphism in *E. stauntonii*. The genetic variation among 825 individuals from 18 natural populations of *E. stauntonii* in Hebei Province of China was analyzed using the 10 polymorphic SSR markers. On the basis of the SSR data, the average number of alleles (*N*_A_), expected heterozygosity (*H*_E_), and Shannon’s information index (I) of the 10 primers pairs were 7.000, 0.478, and 0.688, respectively. Lower gene flow (*N*_*m*_ = 1.252) and high genetic differentiation (*F*_st_ = 0.181) were detected in the populations. Analysis of molecular variance (AMOVA) revealed that most of the variation (81.47%) was within the populations. Integrating the results of STRUCTURE, UPGMA (Unweighted Pair Group Method with Arithmetic Mean) clustering, and principal coordinate analysis, the 825 samples were grouped into two clusters associated with geographical provenance (southwestern and northeastern regions), which was consistent with the results of a Mantel test (*r* = 0.56, *p* < 0.001). Overall, SSR markers developed in related genera were effective to study the genetic structure and genetic diversity in geographical populations of *E. stauntonii*. The results provide a theoretical basis for conservation of genetic resources, genetic improvement, and construction of a core collection for *E. stauntonii*.

## Introduction

Plants provide the fundamental support system for life on Earth and plant diversity is a key property of ecosystems (Moreira et al., 2016). Lamiaceae, with more than 7,000 species around the world, is a large family of flowering plants and an important part of plant diversity (Harley et al., 2004). Some species of the family have been commercially exploited worldwide (e.g., *Lavandula angustifolia*) (Pistelli et al., 2017), while many others are known locally for their ornamental, biological, and medicinal properties (e.g., *Hedeoma patens*) (Leyva-Lopez et al., 2016). *Elsholtzia stauntonii* Benth., a perennial deciduous subshrub of the Lamiaceae family, is widely distributed in China, especially in Hebei Province (Editorial committee of the Flora of China, and Chinese Academy of Sciences, 1977). *Elsholtzia stauntonii* is aromatic and its essential oils show insecticidal activity against certain pests of stored products (Lv et al., 2012). Consequently, *E. stauntonii* is used in novel biological insecticides (Liang et al., 2021). With its dense flowers, late flowering and bright colors, *E. stauntonii* is a popular bedding plant cultivated in public gardens and a wild nectar plant (Tian et al., 2007). Its branches and leaves are also used as a traditional Chinese medicinal material for treating common colds, toothache, and digestive system diseases (Zheng et al., 1999; Pekhova et al., 2020). Furthermore, the species is of ecological importance owing to its strong tolerance of drought and barren environments (Si and Peng, 2017). Previous studies on *E. stauntonii* have mainly focused on its morphological traits (Zhou et al., 2016), cultivation and propagation (Zhou et al., 2014), and extraction and identification of volatile oils (Guo et al., 2012; Xing et al., 2019). Little information is available on the genome and genetic diversity of this species. Studies of genetic diversity have been performed on many Lamiaceae species to clarify the genetic relationships among species (Bahadirli and Ayanoglu, 2020) and to identify molecular markers associated with agronomic traits for marker-assisted breeding (Lim et al., 2021). Therefore, with recognition of the value of this important but neglected species, it is crucial to evaluate the genetic variation in *E. stauntonii*.

Understanding the genetic diversity and structure of germplasm resources is a prerequisite for formulating effective breeding and conservation strategies (Gepts, 2006; Engelhardt et al., 2014; Vanavermaete et al., 2021). In recent years, with advances in molecular biological technologies, molecular marker-based analysis of genetic variation has avoided the limitations of morphological and biochemical indicators that are readily affected by environmental factors, and are more accurate for evaluation of population genetic parameters (Agarwal et al., 2008; Nadeem et al., 2018). Among the various types of molecular markers, microsatellites (or simple sequence repeats; SSRs) have become the preferred choice because of their high degree of reproducibility, ability to identify high levels of genetic polymorphism, codominant inheritance, and abundance in plant genomes (Powell et al., 1996; Grover and Sharma, 2016; Jose et al., 2018). Currently, SSR markers have been widely used in assessments of population genetic diversity (Gadissa et al., 2018), cultivar identification (Pinto et al., 2018; Sulu et al., 2020), and association mapping (Yang et al., 2020) as effective tools to describe genetic variation in plants. Using available genomic or expressed sequence tag databases (Vieira et al., 2016), a large number of SSR markers have been developed in Lamiaceae species, such as *Scutellaria baicalensis* (Zhang et al., 2014), *Perilla frutescens* (Ha et al., 2021), and *Salvia splendens* (Jiao et al., 2020). However, owing to the lack of genetic information, to date no SSR markers are available for evaluation of the genetic diversity within *E. stauntonii*, which limits its breeding and commercial development.

An alternative approach to overcome the time-consuming and costly development of SSR primers for target species is to exploit the transferability of SSR primers between related species or genera (Peakall et al., 1998; Pan et al., 2018). The transfer of SSR markers has been successful in many cases, as documented among Lamiaceae species (Karaca et al., 2013), from *Gossypium hirsutum* and *Corchorus olitorius* to Malvaceae species (Satya et al., 2016), among *Allium* species and among members of the Alliaceae (Barboza et al., 2018), and from *Ricinus communis* to Euphorbiaceae species (Dharajiya et al., 2020).

In this study, cross-genera amplification of SSR markers in Lamiaceae species was used to enrich the genetic database of *E. stauntonii*. On this basis, the genetic diversity and population structure of 825 *E. stauntonii* individuals sampled from 18 regions in Hebei Province, China, which is an important center in the distribution range of the species, were successfully evaluated using 10 highly polymorphic cross-transferable SSR markers. The results provide insight into the genetic resources of *E. stauntonii*, and represent a foundation for the effective management and genetic improvement of the species.

## Materials and Methods

### Plant Materials

A field survey of extant *E. stauntonii* populations in Hebei Province, China was conducted from July to September 2020. Samples of 825 mature and disease-free individuals were randomly collected from 18 populations in the natural distribution area; the sample size per population ranged from 43 to 50 individuals (Table 1). To ensure sufficient geographical representation of the samples, the distance between sampled individuals was more than 50 m. The GPS coordinates were recorded for each population. The altitude of all sampled populations ranged from 540 to 1,200 m, with an average altitude of 937 m. Fresh leaves were desiccated in silica gel and stored at –20°C until use.

**TABLE 1:** Location and sampling site characteristics of 18 *Elsholtzia stauntonii* geographical populations (825 individuals).

Population name	Sample size	Location	Latitude (N)/Longitude (E)	Altitude (m)
HS	50	Shexian, Handan	N:36°36′/E:113°52′	880
XW	44	Wangnaocun, Xingtai	N:36°54′/E:114°4′	540
XM	47	Malingguan, Xingtai	N:37°20/E:113°57′	900
SZ	46	Zanhuangxian, Shijiazhuang	N:37°39′/E:114°23′	969
SP	48	Pingshanxian, Shijiazhuang	N:38°15′/E:114°11′	1,200
BF	46	Fupingxian, Baoding	N:38°53′/E:114°1′	1,200
BZ	46	Zoumayizhen, Baoding	N:39°8′/E:114°36′	915
ZQ	46	Qiaomaichuancun, Zhangjiakou	N:40°2′/E:115°16′	1,200
ZF	47	Feihuyu, Zhangjiakou	N:39°43′/E:114°38′	1,200
BL	43	Laishuixian, Baoding	N:39°23′/E:115°46′	984
BY	47	Yixian, Baoding	N:39°14′/E:114°58′	975
ZC	46	Chichengxian, Zhangjiakou	N:40°58′/E:115°57′	1,000
CF	43	Fengningxian, Chengde	N:41°46′/E:116°20′	1,100
CY	47	Yunwushan, Chengde	N:41°9′/E:116°45′	800
CL	43	Longhuaxian, Chengde	N:41°22′/E:117°48′	800
CX	46	Xinglongxian, Chengde	N:40°25′/E:117°30′	700
CC	46	Chengdexian, Chengde	N:40°46′/E:118°10′	800
QD	44	Dushan, Qinhuangdao	N:40°29′/E:118°48′	700

### DNA Extraction and Genotyping With Simple Sequence Repeats Markers

Total genomic DNA of all samples was extracted using a Plant Genomic DNA Kit (Tiangen Biotech, Beijing, China). The DNA quality was evaluated by 1.0% agarose gel electrophoresis. DNA quantification was conducted with a NanoDrop 2000 spectrophotometer (Thermo Fisher Scientific, Wilmington, DE, United States) and the DNA concentration was adjusted to 20 ng/μL.

In total, 497 SSR markers from eight genera of Lamiaceae were used to perform cross-amplification tests on 16 randomly selected *E. stauntonii* samples from different populations (Table 2). Primers were synthesized by RuiBiotech (Beijing, China). To allow fluorescent labeling, the universal M13 sequence was added to the 5′ end of all forward primers. The PCR mixture was prepared in accordance with the method of Zhang et al. (2015) with modifications. Briefly, the PCR mixture (20 μl) contained 10 μl of 2 × Taq PCR Mix, 2 μl (40 ng) genomic DNA, 4 μl (4 pmol) fluorescent-dye-labeled M13 primer, and 4 μl (4 pmol) mixed complementary forward and reverse primers. The temperature profile used was as follows: an initial step of 5 min at 94°C, followed by 35 cycles of 30 s at 94°C, 30 s at 54°C, and 1 min at 72°C, then eight cycles of 30 s at 94°C, 30 s at 53°C, and 30 s at 72°C, and a final step of 10 min at 72°C. Capillary electrophoresis of the PCR products was performed using an ABI 3730xl DNA Analyzer (Tiangen Biotech, Beijing, China). GeneMarker version 2.2.0 software (Holland and Parson, 2011) was used to analyze the fragment size of the products and determine whether the primers detected polymorphisms.

**TABLE 2:** SSR markers from eight genera in the Lamiaceae used to evaluate cross-genera transferability to *Elsholtzia stauntonii*.

Genera used for cross amplification	Number of SSR markers tested	References
*Salvia* L.	297	Radosavljević et al., 2011, 2012; Jiao et al., 2020; Krak et al., 2020
*Perilla* L.	128	Kwon et al., 2005; Sa et al., 2015, 2018, 2019; Ma et al., 2017; Wang et al., 2017; Park et al., 2019; Ha et al., 2021
*Mentha* L.	25	Vining et al., 2019
*Hyptis* Jacq.	15	Blank et al., 2014
*Rosmarinus* L.	12	Segarra-Moragues and Gleiser, 2009
*Scutellaria* L.	10	Zhang et al., 2014
*Pogostemon* Desf.	6	Sandes et al., 2013
*Leonurus* L.	4	Ren, 2012

### Data Analysis

The SSR markers that successfully amplified single and specific bands within the expected product size range were considered to be transferable. The percentage transferability for each donor genus was calculated as (number of SSRs transferred/total number of SSRs screened) × 100. Similarly, the percentage transferability of SSR markers classified by repeat motifs (di-/tri-/tetra-/penta-/hexa-/complex-nucleotide) was estimated. Population diversity and population structure of *E. stauntonii* were assessed based on the transferred microsatellite loci.

For each SSR locus, important indices of genetic diversity were calculated using GenAlEx version 6.5 (Peakall and Smouse, 2012) and Microsatellite-Toolkit (Park, 2001) software. The indices comprised number of alleles (*N*_A_), number of effective alleles (*N*_E_), Shannon’s information index (I), observed heterozygosity (*H*_O_), expected heterozygosity (*H*_E_), inbreeding coefficient (*F*_is_), gene flow (*N*_m_), and genetic differentiation index (*F*_st_). The polymorphic information content (PIC) value and Hardy–Weinberg equilibrium (HWE) for the loci were calculated using PowerMarker version 3.25 (Liu and Muse, 2005).

Analysis of molecular variance (AMOVA) was conducted using Arlequin version 3.5 (Excoffier and Lischer, 2010) to estimate the variance components of genetic variation among and within populations. Bayesian analysis was performed to evaluate the population genetic structure and detect the most likely number of population genetic clusters of *E. stauntonii* using STRUCTURE version 2.3.4 (Pritchard et al., 2000). For each simulated value of *K* (range from 1 to 18), 15 independent runs were performed with a burn-in period of 200,000 iterations followed by 1,000,000 Markov chain Monte Carlo repetitions. The Δ*K* method (Evanno et al., 2005) was implemented in the Structure Harvester (Earl and VonHoldt, 2012) program based on the STRUCTURE results to determine the optimum *K* value. The percentage membership of each individual in every cluster (*Q* value) was determined; an individual with a *Q* value higher than 0.80 was considered to have a single genetic component (a pure individual). Nei’s genetic distance (DA) (Nei et al., 1983) among the 825 individuals and 18 populations was calculated using PowerMarker version 3.25 (Liu and Muse, 2005). To explore the genetic relationships among all samples, the DA matrix was used to construct a dendrogram by hierarchical clustering with the unweighted pair group method with arithmetic mean (UPGMA) using PowerMarker version 3.25 (Liu and Muse, 2005). The dendrograms were visualized, manipulated, and annotated with the Interactive Tree of Life online tool (Letunic and Bork, 2007). Based on the standardized covariance of genetic distance, a principal coordinate analysis (PCoA) was conducted with GenAlEx version 6.5 (Peakall and Smouse, 2012). A clustering heatmap was prepared with Microsoft Excel based on the pairwise Nei’s genetic distance between populations and the *F*_st_ values.

The geographic distance between the sampling locations was calculated with Geographic Distance Matrix Generator version 1.2.3 software^1^. The Mantel test was performed to analyze the correlation between geographic and genetic distance of different populations using the “ggpubr^2^” and “ggplot2” (Ginestet, 2011) packages in R version 4.1.1 (Ihaka and Gentleman, 1996). The map was created using the “sf” (Pebesma, 2018), “ggplot2” (Ginestet, 2011), and “ggspatial^3^” packages in R version 4.1.1.

## Results

### Transferability of Simple Sequence Repeats Markers

**FIGURE 1:**
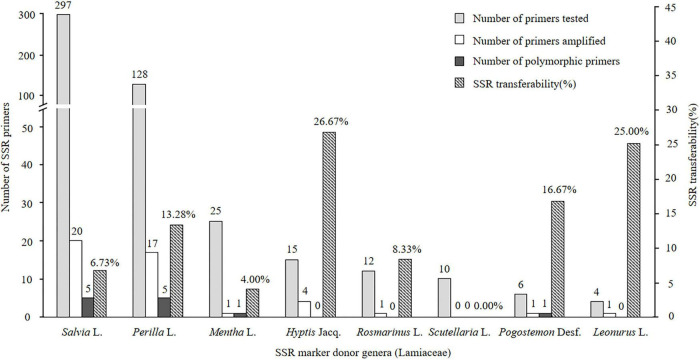
Confamiliar transferability of SSR markers to *Elsholtzia stauntonii* from other genera of Lamiaceae.

**FIGURE 2:**
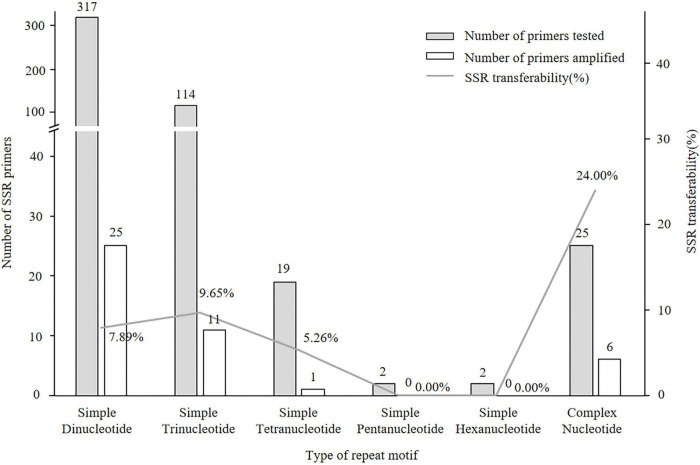
Transferability of SSR markers to *Elsholtzia stauntonii* based on SSR repeat motif length. The repeat motif of eighteen SSRs was not available.

Of the 497 SSRs mined, 45 primers (9.05%) successfully amplified genomic DNA of *E. stauntonii* and produced PCR products of the expected size (Supplementary Table 1). Transferability of the SSR markers varied among eight genera of Lamiaceae: 26.67% of the SSR markers of *Hyptis* Jacq., 25.00% of *Leonurus* L., 16.67% of *Pogostemon* Desf., 13.28% of *Perilla* L., 8.33% of *Rosmarinus* L., 6.73% of *Salvia* L., and 4.00% of *Mentha* L. were transferable to *E. stauntonii*, whereas no SSR marker was transferable from *Scutellaria* L. (Figure 1). The transferability of SSR markers based on simple di-/tri-nucleotide repeat motifs (7.89–9.65%) was higher than that based on tetra-/penta-/hexa-nucleotide repeat motifs (Figure 2). The SSR markers based on complex (simple imperfect, compound perfect/imperfect) nucleotide repeats also showed high transferability (24.00%).

### Polymorphism Analysis Based on Transferable Simple Sequence Repeats Markers

Among the 45 transferable SSR markers, 35 markers were monomorphic (33 primers) or showed very low levels of polymorphism (two primers) based on capillary electrophoresis results (Supplementary Table 1). Ten (2.01%) primer pairs showed higher polymorphism and were used for analysis of genetic diversity in *E. stauntonii* (Table 3).

**TABLE 3:** Characterization of 10 polymorphic SSR primer pairs.

SSR primer	Primer sequence (5′ to 3′)	Repeat motif	Allele size (bp)	*N*_A_	*H*_O_	*H*_E_	PIC	*F*_is_	HWE
KNUPF15^a^	F:CCACACGTAAACCTCATAAACC R:TTATCTCTAAAGAAATCGGGCA	(CT)_16_	168–186	10	0.611	0.750	0.708	0.185	***
KNUPF67^b^	F:ATTGATTCTCTATCAACCTGGC R:CTCATCATCGGATCAACCTAGT	(GCT)_7_	189–201	4	0.194	0.264	0.230	0.265	***
ssps251^c^	F:GACGCTCAAATGGTGAATCC R:ACGCTGATCTGGAAAGATGG	(TC)_11_	231–277	24	0.469	0.879	0.868	0.466	***
ssps321^c^	F:ACGTGAACACACACCACCAT R:ACAGCATCAGCAACAGCAAC	(GCT)_10_	220–235	8	0.336	0.425	0.390	0.209	***
ssps344^c^	F:CCTTCACCTGGGATGGAGTA R:GCCCTTTCAACCAAAACAAA	(CT)_10_	177–235	2	0.241	0.214	0.191	−0.126	***
ssps512^c^	F:GGCTCCTCGTTTTATGGTGA R:GCATTAGTTGATGCCGAGGT	(GA)_11_	163–195	3	0.236	0.499	0.376	0.527	***
ssps711^c^	F:CCGACGTGAACATACACCAC R:TTATGCAGCAGCAGGTTTTG	(GCT)_10_	335–350	6	0.327	0.414	0.379	0.210	***
Pca6^d^	F: ACAAAGGGTTGACGATTG R:GTGATGAAACTGTCTCTCCTG	(TG)_4_ (TC) (TG)_5_ (AG)_4_.(TGTT)_3_	232–248	9	0.391	0.584	0.545	0.330	***
GBPF203^e^	F:GTTTTGTTGCAGCTCGATTT R:TGGGTTTGGAAAGTATTGATG	(GA)_5_ (TAA) (AG)_26_	132–162	2	0.439	0.352	0.290	−0.247	***
C3787^f^	F:GAGAGTACGGCGAGTAATTG R:TATCAACGTGAAGGAGACTTG	(ATTT)_4_	145–157	2	0.356	0.396	0.317	0.101	**
Mean				7.000	0.360	0.478	0.429	0.192	–

N_A_, number of alleles; H_O_, observed heterozygosity; H_E_, expected heterozygosity; PIC, polymorphic information content; F_is_, inbreeding coefficient; HWE, Hardy–Weinberg equilibrium; **p < 0.01; ***p < 0.001. ^a, b, c, d, e, f^, Previously published microsatellite markers (^a^: Sa et al., 2018; ^b^: Sa et al., 2019; ^c^: Jiao et al., 2020; ^d^: Sandes et al., 2013; ^e^: Sa et al., 2015; ^f^: Vining et al., 2019).

In total, 70 alleles were amplified by the 10 polymorphic SSR markers among 825 *E. stauntonii* individuals, with a mean of 7.000 observed alleles per locus, and ranging in length from 132 to 350 bp (Table 3). The number of alleles per marker varied from 2 (ssps344, GBPF203, and C3787) to 24 (ssps251). Among the 10 markers, the observed heterozygosity (*H*_O_) of eight markers was lower than the expected heterozygosity (*H*_E_), and the average inbreeding coefficient (*F*_is_) was positive. The PIC values ranged from 0.191 to 0.868, with a mean of 0.429. Eight SSR markers showed moderate or high polymorphism levels (PIC > 0.25) and two markers (ssps344 and KNUPF67) showed low polymorphism levels (PIC < 0.25) among the tested *E. stauntonii* populations. All SSR genotyping data for the 10 loci showed strongly significant deviation from the HWE (*p* < 0.01) (Table 3).

### Population Genetic Diversity and Differentiation

In general, the difference in genetic diversity among the 18 geographical populations of *E. stauntonii* was observed (Table 4). The genetic diversity indices *N*_A_ and *N*_E_ of individual populations ranged from 2.400 (XW) to 3.900 (XM), and from 1.406 (ZQ) to 2.532 (CX), with means of 3.267 and 1.971, respectively. The average *H*_O_ and *H*_E_ were 0.359 and 0.388, ranging from 0.253 (XW) to 0.478 (CX), and from 0.260 (ZQ) to 0.506 (CX), respectively. The I value ranged from 0.450 (ZQ) to 0.887 (CX), with an average of 0.688. The highest level of genetic diversity was detected in CX, whereas XW and ZQ showed the lowest genetic diversity (Table 4).

**TABLE 4:** Genetic diversity for 10 polymorphic SSR markers in 18 natural populations of *Elsholtzia stauntonii* (total of 825 individuals).

Population	*N*_A_	*N*_E_	I	*H*_O_	*H*_E_	*F*_st_	*N*_m_
HS	2.600	1.674	0.520	0.263	0.311		
XW	2.400	1.545	0.477	0.253	0.284		
XM	3.900	2.331	0.699	0.351	0.361		
SZ	3.700	2.030	0.675	0.291	0.358		
SP	3.600	1.969	0.704	0.419	0.393		
BF	3.600	2.066	0.691	0.324	0.353		
BZ	3.600	2.205	0.745	0.377	0.405		
ZQ	2.600	1.406	0.450	0.273	0.260		
ZF	3.700	1.943	0.757	0.417	0.415		
BL	3.100	1.888	0.695	0.416	0.406		
BY	3.300	2.275	0.790	0.438	0.444		
ZC	3.700	2.005	0.775	0.372	0.447		
CF	3.200	1.726	0.624	0.319	0.352		
CY	3.200	2.227	0.848	0.409	0.497		
CL	3.500	1.816	0.608	0.291	0.317		
CX	3.500	2.532	0.887	0.478	0.506		
CC	2.800	2.065	0.782	0.430	0.478		
QD	2.800	1.771	0.664	0.348	0.401		
Mean	3.267	1.971	0.688	0.359	0.388	0.181	1.252

*N_E_, number of effective alleles; I, Shannon’s Information Index; F_st_, genetic differentiation index; N_m_, gene flow.*

The average *F*_st_, a measure of the degree of genetic differentiation among populations, was 0.181 and the average *N*_m_ was 1.252 (Table 4). These results were consistent with those of AMOVA, which revealed that 18.53% of the genetic variation occurred among the populations and 81.47% was present within the populations (Table 5).

**TABLE 5:** Analysis of molecular variance (AMOVA) for 825 *Elsholtzia stauntonii* individuals clustered in 18 populations.

Source of variation	Degrees of freedom	Sum of squares	Percentage of variation (%)	*P*-value
Among populations	17	727.551	18.53%	<0.001
Within populations	807	3178.441	81.47%	<0.001
Total	824	3905.992	100.00%	

**FIGURE 3:**
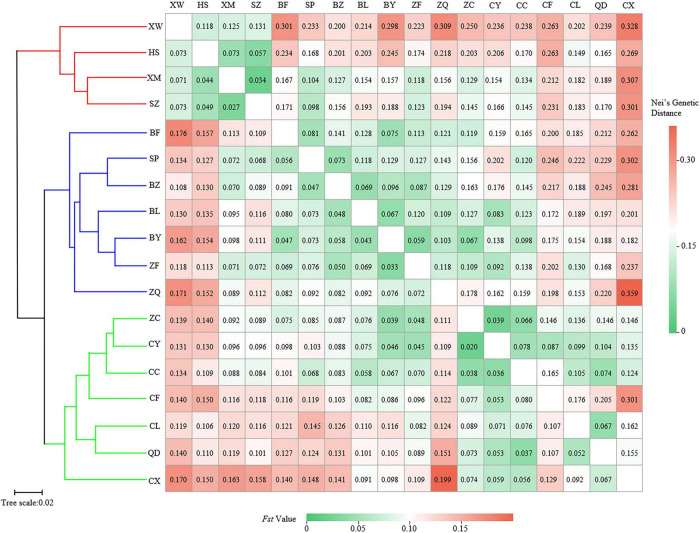
Clustering heatmap of pairwise genetic differentiation index (*F*_st_) (values below the diagonal), and pairwise Nei’s genetic distance (values above the diagonal) among 18 populations of *Elsholtzia stauntonii*.

**FIGURE 4:**
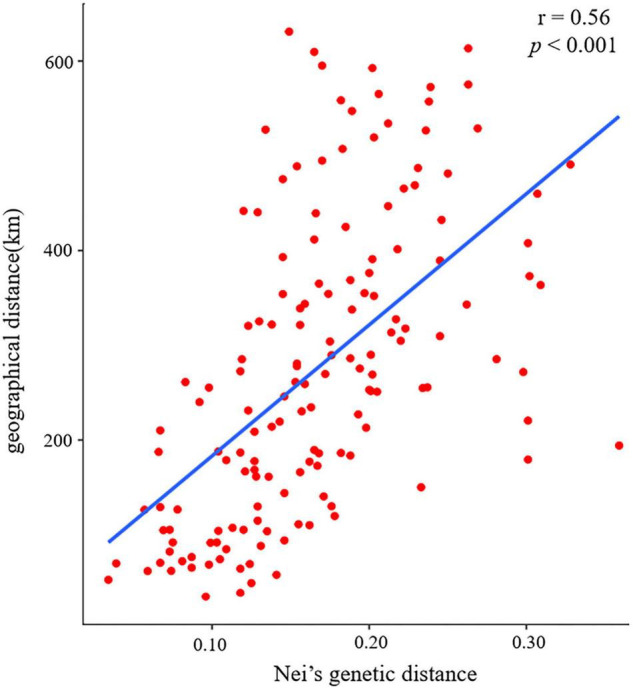
Mantel test results of the correlation between Nei’s genetic distance and geographical distance among 18 populations of *Elsholtzia stauntonii*.

The pairwise *F*_st_ ranged from 0.020 (between populations CY and ZC) to 0.199 (between populations CX and ZQ) (Figure 3). The pairwise Nei’s genetic distance ranged from 0.034 to 0.359, with the highest value (0.359) between the CX and ZQ populations and the second highest (0.328) between the CX and XW populations (Figure 3). In general, populations that were geographically closer had lower genetic distances (SZ–XM, 52.43 km, 0.034), whereas higher genetic distances were observed for populations that were farther apart (CX–XW, 490.81 km, 0.328). Mantel test results revealed a strongly significant correlation between genetic and geographic distance among the 18 natural populations (*r* = 0.56, *p* < 0.001) (Figure 4).

### Genetic Relationship and Population Structure

**FIGURE 5:**
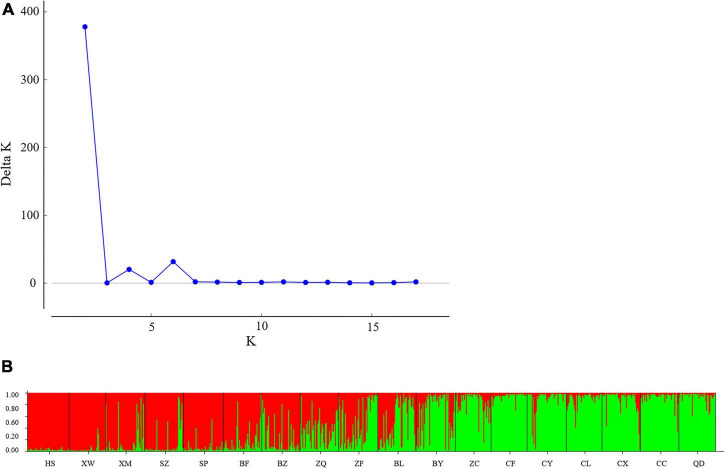
Population structure of 825 *Elsholtzia stauntonii* individuals based on 10 SSR markers. **(A)** Delta *K* values from STRUCTURE analysis of the *E. stauntonii* individuals. **(B)** Histogram from STRUCTURE analysis for the model with *K* = 2.

**FIGURE 6:**
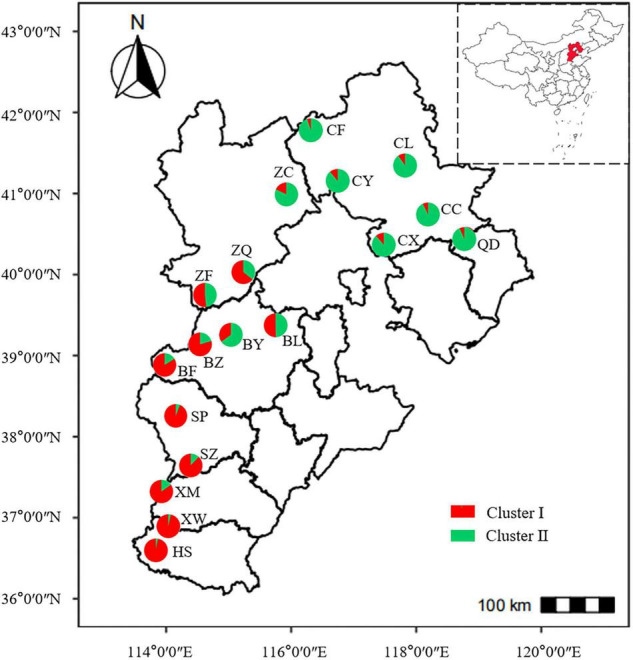
Geographical distribution of 18 populations of *Elsholtzia stauntonii* and lineage contribution to gene pools. The pie diagram was drawn based on the results of STRUCTURE analysis with *K* = 2 and represents the average proportion of cluster membership of all individuals in each population.

To determine the genetic structure of *E. stauntonii* populations, the co-ancestral relationship among populations was analyzed based on a Bayesian assignment model. The *Q* value of 680 individuals, accounting for 82.42% of the total samples, was greater than 0.8, indicating that most individuals had a relatively simple genetic background (Supplementary Table 2). STRUCTURE analysis revealed a distinct peak for Δ*K* at *K* = 2, indicating that the *E. stauntonii* samples could be grouped into two major clusters; these two subpopulations roughly corresponded to the southwestern and northeastern regions of Hebei Province (Figures 5A,B, 6). The four populations (ZQ, ZF, BY, and BL) situated at the junction of the two regions contained abundant genetic variation and the lineages were obviously mixed (Figure 6). These populations contained genetic information of dual origins, with an average assignment of 49.7% to Cluster I (red) and 50.3% to Cluster II (green), whereas the other populations were relatively independent (Figure 6 and Supplementary Table 3).

**FIGURE 7:**
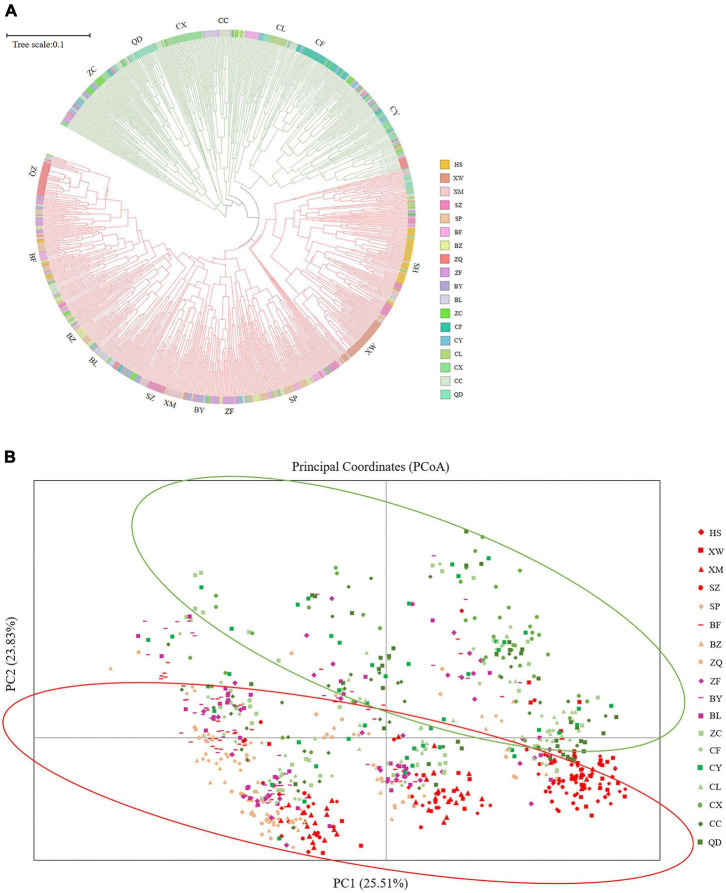
**(A)** UPGMA tree constructed based on Nei’s genetic distance of 825 *Elsholtzia stauntonii* individuals. **(B)** Principal coordinate analysis (PCoA) of 825 individuals from 18 populations.

The UPGMA dendrogram grouped the 18 *E. stauntonii* populations into three major clusters. The populations XW, HS, XM, and SZ formed one cluster, the populations BF, SP, BZ, BL, BY, ZF, and ZQ formed a separate cluster, and the remaining populations (ZC, CY, CC, CF, CL, QD, and CX) formed a third group (Figure 3). However, the UPGMA tree of 825 individuals contained clusters of individuals from different populations and the samples were grouped into two major clades (Figure 7A). Consistent with the population dendrogram, the individuals of seven populations (ZC, CY, CC, CF, CL, QD, and CX) in the northeastern region were merged into a cluster and showed a broadly mixed ancestry, whereas the majority of individuals from the 11 populations in the southwestern region were grouped in the same cluster. In general, genetic proximity was observed between neighboring populations, whereas few genotypes shared an ancestry with individuals in geographically distant populations (Figure 7A).

Principal coordinate analysis was performed to visualize the multidimensional relationships among individuals based on genetic distance (Figure 7B). The closer they were on the PCoA graph, the less genetically differentiated they were, such as the HS, XM and SZ populations. Principal coordinates (PC) 1 and 2 explained 25.51% and 23.83% of the variance, respectively, and a combined 49.34% of the total variation. The PCoA analysis separated the populations into two clusters, which further confirmed the presence of genetic structure among *E. stauntonii* populations (Figure 7B).

## Discussion

### Cross-Genera Transferability of Microsatellite Markers

Cross-taxa transferability enables the development of microsatellite markers, based on markers from related species, at low cost (Kalia et al., 2011; Bernardes et al., 2021). In the present study, the transferability observed (9.05%) was similar to the average of approximately 10% reported in cross-genera transferability studies of eudicots between 1997 and mid-2006 (Barbará et al., 2007). However, the percentage transferability determined here was lower than the cross-genera amplification percentage observed in some families, such as Bignoniaceae (40.58%) (Kalia et al., 2020) and Cactaceae (35.16%) (Bombonato et al., 2019), and much lower than that between species within the same genus (Miranda et al., 2020; Pern et al., 2020; Li et al., 2021). The transferability of SSR markers between species or genera is determined by the conservation of DNA sequences and the stability of primer binding sites in flanking regions of SSRs during evolution (Ellegren, 2000; Saeed et al., 2016). A higher cross-taxa amplification percentage of SSR markers is expected between species with a close phylogenetic relationship, especially when both donor and target species are congeneric (Lesser et al., 2012; Karaca et al., 2013; Bharti et al., 2018). Therefore, it was speculated that *E. stauntonii* might have a relatively distant genetic relationship with its donor species (Jarne and Lagoda, 1996; Li et al., 2017). Nevertheless, although the primary contributing factor is phylogenetic proximity, transferability may also be influenced by other factors, such as the size and complexity of the relevant genome and whether the microsatellite is sited within a coding region (Oliveira et al., 2006).

The SSR transferability is also affected by the base repeat type of SSR core units (Gao et al., 2013). In general, SSR markers with a trinucleotide repeat motif show higher transferability than those with dinucleotide-type repeat motifs (Ellis and Burke, 2007; Harijan et al., 2017). A similar trend in heterologous SSR marker transferability has been reported for Euphorbiaceae, with the transfer rate of simple trinucleotide repeats being 11.44% higher than that of dinucleotide repeats (Dharajiya et al., 2020). One reason might be that the selection of frameshift mutations limits the fixation of microsatellites whose motif lengths are not divisible by three (e.g., di-/tetra-/pentanucleotide repeats), whereas fixation of mutant microsatellite whose motif lengths are divisible by three (tri-/hexanucleotide repeats) is not affected by differential selection pressures in coding and non-coding regions (Metzgar et al., 2000; Dutta et al., 2011; Shivakumar et al., 2017). Moreover, microsatellites based on a complex nucleotide sequence show a lower mutation rate and higher transferability than simple repeats (Jin et al., 1996; Kalia et al., 2020). Given that more than 63% of the SSR markers used in the present study were di-nucleotide motif repeats, this may be an additional reason for the low transferability. Regardless, the 10 polymorphic transferable SSR markers identified in this study could be used to accelerate analysis of the genetic diversity of *E. stauntonii* and related species.

### Genetic Diversity and Population Structure Analysis

All 10 transferable SSR primers used in the present study deviated significantly from the HWE (*p* < 0.01), which might reflect that the samples were collected from natural populations affected by complex environmental factors and human activities (Wang and Shete, 2012; Peng et al., 2021). The investigation found that *E. stauntonii* mainly resided beside rivers and valleys at lower altitudes, and its living environment was easily disturbed by tourism, grazing, and farmland planting. The mean PIC was 0.429, indicating that the genetic diversity of *E. stauntonii* germplasm in Hebei Province was generally moderate (0.25 < PIC < 0.5) (Botstein et al., 1980), which was lower than that reported for *Scutellaria baicalensis* (0.72) (Qi et al., 2015) and *Perilla frutescens* (0.582) (Park et al., 2019). The genetic diversity possessed by a species is determined by its evolutionary and ecological history (Aboukhalid et al., 2017). For *E. stauntonii*, the wide original geographic distribution theoretically contributes to the high level of genetic diversity detected. The moderate genetic diversity observed in this study might be limited by the number of markers, the test method, or the regions of sample collection (Budak et al., 2004; Serrote et al., 2020). Furthermore, it is generally accepted that markers of high transferability are less polymorphic among closely related species (Ni et al., 2015).

Genetic variation of a population can be measured by the number of alleles and heterozygosity (Huang et al., 2020; Savić et al., 2021). The mean *N*_A_ (7.000) and *N*_E_ (1.971) observed in the present study were slightly higher than the values (6.45 and 1.67, respectively) for 12 *Plectranthus edulis* populations evaluated using 20 SSR markers (Gadissa et al., 2018), but lower than the values reported for *Salvia splendens* (*N*_A_ = 11.829, *N*_E_ = 3.261) (Jiao et al., 2020) and *Juglans nigra* (*N*_A_ = 9.99, *N*_E_ = 7.51) (Schneider et al., 2019). Compared with previous studies on Lamiaceae species, *E. stauntonii* (*H*_O_ = 0.360) has higher genetic diversity than *Salvia fruticosa* (*H*_O_ = 0.307) (Radosavljević et al., 2015) and *Hedeoma piperita* (*H*_O_ = 0.3) (Herrera-Arroyo et al., 2020), but a higher level of genetic diversity has been observed in *Rosmarinus officinalis* (*H*_O_ = 0.63) (Segarra-Moragues et al., 2015). These results also indicated that the tested *E. stauntonii* samples showed moderate genetic diversity. The observed heterozygosity in the present study was lower than the expected heterozygosity (*H*_E_ = 0.478), indicating that the number of heterozygotes was lower than the predicted value (García-Arias et al., 2018). The average inbreeding coefficient (*F*_is_ = 0.192) in the population was positive and high, which similarly suggested there was an overabundance of homozygotes (Liu et al., 2019; Bairu et al., 2021). The reproductive characteristics of species might be an important factor in heterozygote deficiency in populations; for example, the combination of hermaphroditic flowers and profuse flowering of *E. stauntonii* may lead to improved levels of inbreeding which can reduce the genetic diversity of this widely distributed species (Zigene et al., 2021).

Genetic differentiation is an important index of the genetic structure of populations within a species (Wang, 2020). In the current study, gene flow of *E. stauntonii* (*N*_m_ = 1.252) was moderately low, lower than that reported for *Eragrostis tef* (*N*_m_ = 4.742) (Tadesse et al., 2021) and *Mucuna pruriens* (*N*_m_ = 7.48) (Lepcha and Sathyanarayana, 2021), but it was consistent with the high genetic differentiation (*F*_st_ = 0.181) (Balloux and Lugon-Moulin, 2002). The low gene flow, which might be caused by the small sampling range and habitat fragmentation, increased the within-population genetic variation and promoted population differentiation (Young et al., 1996; Li et al., 2014; Peng et al., 2021). Notably, among the 18 populations, the greatest genetic differentiation was observed between the CX and ZQ populations, which may be considered as potential parents for hybrid breeding. Furthermore, AMOVA revealed that the variation among *E. stauntonii* populations (18.53%) was higher than that of many other plant species, such as *Origanum vulgare* subsp. *hirtum* (8%) (Alekseeva et al., 2021) and *Allium sativum* L. (12.29%) (Poljuha et al., 2021), suggesting that disruption of gene flow between the populations played a role in determining the genetic differentiation of populations. Similarly, the results of the Mantel test (*r* = 0.56, *p* < 0.001) showed that increase in geographic distance reduced gene flow. Therefore, geographic distance may be the principal factor for genetic differentiation in *E. stauntonii*.

In the UPGMA clustering analysis, a certain deviation was observed in the clustering of individuals and of populations, which might be due to the high degree of gene exchange between populations. Overall, the clustering results observed with STRUCTURE, PCoA, and UPGMA were essentially similar, indicating that there might be two gene pools of *E. stauntonii*. Examination of the topography and geomorphology in Hebei Province indicated that the genetic structure of *E. stauntonii* was delimited according to geographical location and topographic barriers: one genealogical lineage each was located near the Taihang Mountains and the Yanshan Mountains. Previous studies have shown that geographical barriers play a crucial role in preventing plant dispersal (Fan et al., 2013; Zhao and Gong, 2015; Zeng et al., 2018), for example, the Tanaka Line in southwest China has a marked effect on the divergence and evolution of plant species (Ju et al., 2018). In this study, populations ZQ and CX on both sides of the mountain ranges presented the greatest *F*_st_ and genetic distance values, although their geographic distance was relatively close (194.14 km apart from each other), which also indicated the role of geographical barriers in genetic differentiation. The Taihang and Yanshan mountains may be responsible for maintaining the major southwest–northeast split of *E. stauntonii* populations by hindering animal-mediated gene exchange. Thus, natural adaptation and geographical barriers may explain the differentiation of the two regions. Moreover, in the present analyses, each group was more or less intermingled with lineages of the other group, indicating that there was a certain level of gene flow between the populations, which has also been reported in *Tectona grandis* (Balakrishnan et al., 2021). The levels of genetic admixture differed substantially from population to population and seemed to be associated with the geographic location of the population, which indicated that interbreeding was an ongoing process, especially for the ZF, ZQ, BY, and BL populations (Mangaravite et al., 2016). These four populations had relatively high gene introgression, indicating that these populations had relatively rich genetic backgrounds and might be potential centers of diversification in this species. From the perspective of cultivar development and conservation, these populations may be valuable because they may contain novel alleles.

## Conclusion

In this study, SSR markers from seven genera of Lamiaceae were transferable to *E. stauntonii*. Analysis based on 10 transferable SSR markers showed that, although the genetic diversity in 18 geographical populations of *E. stauntonii* was slightly lower than that of certain other species in the Lamiaceae, the genetic diversity was generally rich and the populations showed a high degree of genetic differentiation. STRUCTURE, PCoA, and UPGMA clustering analyses of all individuals resolved two genetic clusters, which might reflect geographical barriers to gene flow. By screening the study materials with superior traits and rich genetic variation, some individuals in the present experiment are expected to be utilized in the future improvement programmes of *E. stauntonii* to meet the increasing demand from the floriculture, essential oil, pharmaceutical, and other industries. The information obtained from the analysis of genetic diversity and population structure provides a foundation for the conservation, management, and utilization of *E. stauntonii*.

## Data Availability Statement

The original contributions presented in the study are included in the article/Supplementary Material, further inquiries can be directed to the corresponding authors.

## Author Contributions

YL and BC conceived and designed the experiments and obtained the funding. CZ, XL, and HZ conducted the experiments. CJ and BC contributed to the investigation. CZ, LH, and ML collected and analyzed the data. CZ wrote the manuscript. YL provided suggestions and comments for the manuscript. All authors read and approved the final manuscript.

## Conflict of Interest

The authors declare that the research was conducted in the absence of any commercial or financial relationships that could be construed as a potential conflict of interest.

## Publisher’s Note

All claims expressed in this article are solely those of the authors and do not necessarily represent those of their affiliated organizations, or those of the publisher, the editors and the reviewers. Any product that may be evaluated in this article, or claim that may be made by its manufacturer, is not guaranteed or endorsed by the publisher.

## Footnotes

1
http://biodiversityinformatics.amnh.org/opensource/gdmg/index.php

2
https://cran.r-project.org/package=ggpubr

3
https://cran.r-project.org/package=ggspatial

## References

[B1] Aboukhalid K. Machon N. Lambourdiere J. Abdelkrim J. Bakha M. Douaik A. (2017). Analysis of genetic diversity and population structure of the endangered *Origanum compactum* from Morocco, using SSR markers: implication for conservation. *Biol. Conserv.* 212 172–182. 10.1016/j.biocon.2017.05.030

[B2] Agarwal M. Shrivastava N. Padh H. (2008). Advances in molecular marker techniques and their applications in plant sciences. *Plant Cell Rep.* 27 617–631. 10.1007/s00299-008-0507-z 18246355

[B3] Alekseeva M. Zagorcheva T. Rusanova M. Rusanov K. Atanassov I. (2021). Genetic and flower volatile diversity in natural populations of *Origanum vulgare* subsp. hirtum (Link) Ietsw. in Bulgaria: toward the development of a core collection. *Front. Plant Sci.* 12:679063. 10.3389/fpls.2021.679063 34335650 PMC8320660

[B4] Bahadirli N. P. Ayanoglu F. (2020). Genetic diversity of *Salvia* species from Turkey assessed by microsatellite markers. *J. Appl. Res. Med. Aromat. Plants* 20:100281. 10.1016/j.jarmap.2020.100281

[B5] Bairu M. W. Amelework A. B. Coetzer W. G. (2021). Genetic diversity and population structure of six South African *Acacia mearnsii* breeding populations based on SSR markers. *J. Plant Res.* 134 1243–1252. 10.1007/s10265-021-01331-2 34302570

[B6] Balakrishnan S. Dev S. A. Sakthi A. R. Vikashini B. Bhasker T. R. Magesh N. S. (2021). Gene-ecological zonation and population genetic structure of *Tectona grandis* L.f. in India revealed by genome-wide SSR markers. *Tree Genet. Genomes* 17:33. 10.1007/s11295-021-01514-x

[B7] Balloux F. Lugon-Moulin N. (2002). The estimation of population differentiation with microsatellite markers. *Mol. Ecol.* 11 155–165. 10.1046/j.0962-1083.2001.01436.x 11856418

[B8] Barbará T. Palma-Silva C. Paggi G. M. Bered F. Fay M. F. Lexer C. (2007). Cross-species transfer of nuclear microsatellite markers: potential and limitations. *Mol. Ecol.* 16 3759–3767. 10.1111/j.1365-294X.2007.03439.x 17850543

[B9] Barboza K. Beretta V. Kozub P. C. Salinas C. Morgenfeld M. M. Galmarini C. R. (2018). Microsatellite analysis and marker development in garlic: distribution in EST sequence, genetic diversity analysis, and marker transferability across *Alliaceae*. *Mol. Genet. Genomics* 293 1091–1106. 10.1007/s00438-018-1442-5 29705936

[B10] Bernardes V. Murakami D. M. Bizão N. Souza T. N. Silva M. J. Telles M. P. C. (2021). Transferability and characterization of microsatellite markers from *Byrsonima cydoniifolia* A. Juss. (*MALPIGHIACEAE*) in seven related taxa from Cerrado biome reveal genetic relationships. *Mol. Biol. Rep.* 48 4039–4046. 10.1007/s11033-021-06411-z 34014470

[B11] Bharti R. Kumar S. Parekh M. J. (2018). Development of genomic simple sequence repeat (gSSR) markers in cumin and their application in diversity analyses and cross-transferability. *Ind. Crops Prod.* 111 158–164. 10.1016/j.indcrop.2017.10.018

[B12] Blank A. F. Jesus A. S. Santos C. P. Grando C. Pinheiro J. B. Zucchi M. I. (2014). Development and characterization of novel microsatellite markers in *Hyptis pectinate* (*Lamiaceae*). *Genet. Mol. Res.* 13 10173–10176. 10.4238/2014.december.4.11 25501228

[B13] Bombonato J. R. Bonatelli I. A. S. Silva R. G. A. Moraes E. M. Zappi D. C. Taylor N. P. (2019). Cross-genera SSR transferability in cacti revealed by a case study using Cereus (*Cereeae*, *Cactaceae*). *Genet. Mol. Biol.* 42 87–94. 10.1590/1678-4685-GMB-2017-0293 30794719 PMC6428128

[B14] Botstein D. White R. L. Skolnick M. Davis R. W. (1980). Construction of a genetic linkage map in man using restriction fragment length polymorphisms. *Am. J. Hum. Genet.* 32 314–331. 6247908 PMC1686077

[B15] Budak H. Shearman R. C. Parmaksiz I. Dweikat I. (2004). Comparative analysis of seeded and vegetative biotype buffalograsses based on phylogenetic relationship using ISSRs, SSRs, RAPDs, and SRAPs. *Theor. Appl. Genet.* 109 280–288. 10.1007/s00122-004-1630-z 15024466

[B16] Dharajiya D. T. Shah A. Galvadiya B. P. Patel M. P. Srivastava R. Pagi N. K. (2020). Genome-wide microsatellite markers in castor (*Ricinus communis* L.): identification, development, characterization, and transferability in *Euphorbiaceae*. *Ind. Crops Prod.* 151:112461. 10.1016/j.indcrop.2020.112461

[B17] Dutta S. Kumawat G. Singh B. P. Gupta D. K. Singh S. Dogra V. (2011). Development of genic-SSR markers by deep transcriptome sequencing in pigeonpea [*Cajanus cajan* (L.) Millspaugh]. *BMC Plant Biol.* 11:17. 10.1186/1471-2229-11-17 21251263 PMC3036606

[B18] Earl D. A. VonHoldt B. M. (2012). STRUCTURE HARVESTER: a website and program for visualizing STRUCTURE output and implementing the Evanno method. *Conserv. Genet. Resour.* 4 359–361. 10.1007/s12686-011-9548-7

[B19] Editorial committee of the Flora of China, and Chinese Academy of Sciences (1977). “*Elsholtzia*(*Lamiaceae*),” in *Flora of China*, Vol. 66 eds Wu Z. Y. Li X. W. (Beijing: Science Press). 304–348.

[B20] Ellegren H. (2000). Microsatellite mutations in the germline: implications for evolutionary inference. *Trends Genet.* 16 551–558. 10.1016/S0168-9525(00)02139-9 11102705

[B21] Ellis J. R. Burke J. M. (2007). EST-SSRs as a resource for population genetic analyses. *Heredity* 99 125–132. 10.1038/sj.hdy.6801001 17519965

[B22] Engelhardt K. A. M. Lloyd M. W. Neel M. C. (2014). Effects of genetic diversity on conservation and restoration potential at individual, population, and regional scales. *Biol. Conserv.* 179 6–16. 10.1016/j.biocon.2014.08.011

[B23] Evanno G. Regnaut S. Goudet J. (2005). Detecting the number of clusters of individuals using the software STRUCTURE: a simulation study. *Mol. Ecol.* 14 2611–2620. 10.1111/j.1365-294X.2005.02553.x 15969739

[B24] Excoffier L. Lischer H. E. L. (2010). Arlequin suite ver 3.5: a new series of programs to perform population genetics analyses under Linux and Windows. *Mol. Ecol. Resour.* 10 564–567. 10.1111/j.1755-0998.2010.02847.x 21565059

[B25] Fan D. M. Yue J. P. Nie Z. L. Li Z. M. Comes H. P. Sun H. (2013). Phylogeography of *Sophora davidii* (*Leguminosae*) across the ‘Tanaka-Kaiyong Line’, an important phytogeographic boundary in Southwest China. *Mol. Ecol.* 22 4270–4288. 10.1111/mec.12388 23927411

[B26] Gadissa F. Tesfaye K. Dagne K. Geleta M. (2018). Genetic diversity and population structure analyses of *Plectranthus edulis* (Vatke) Agnew collections from diverse agro-ecologies in Ethiopia using newly developed EST-SSRs marker system. *BMC Genet.* 19:92. 10.1186/s12863-018-0682-z 30309314 PMC6182789

[B27] Gao C. H. Ren X. D. Mason A. S. Li J. Wang W. Xiao M. L. (2013). Revisiting an important component of plant genomes: microsatellites. *Funct. Plant Biol.* 40 645–661. 10.1071/FP12325 32481138

[B28] García-Arias F. L. Osorio-Guarín J. A. Zarantes V. M. N. (2018). Association study reveals novel genes related to yield and quality of fruit in cape gooseberry (*Physalis peruviana* L.). *Front. Plant Sci.* 9:362. 10.3389/fpls.2018.00362 29616069 PMC5869928

[B29] Gepts P. (2006). Plant genetic resources conservation and utilization. *Crop Sci.* 46 2278–2292. 10.2135/cropsci2006.03.0169gas

[B30] Ginestet C. (2011). Ggplot2: elegant graphics for data analysis. *J. R. Stat. Soc. A Stat.* 174 245–245. 10.1111/j.1467-985X.2010.00676_9.x

[B31] Grover A. Sharma P. C. (2016). Development and use of molecular markers: past and present. *Crit. Rev. Biotechnol.* 36 290–302. 10.3109/07388551.2014.959891 25430893

[B32] Guo Z. Q. Liu Z. Z. Wang X. H. Liu W. R. Jiang R. Cheng R. Y. (2012). Elsholtzia: phytochemistry and biological activities. *Chem. Cent. J.* 6:147. 10.1186/1752-153X-6-147 23216850 PMC3536681

[B33] Ha Y. J. Sa K. J. Lee J. K. (2021). Identifying SSR markers associated with seed characteristics in Perilla (*Perilla frutescens* L.). *Physiol. Mol. Biol. Plants* 27 93–105. 10.1007/s12298-021-00933-3 33627965 PMC7873175

[B34] Harijan Y. Nishanth G. K. Katageri I. S. Khadi B. M. (2017). Transferability of heterologous SSR markers to cotton genotypes. *Electron. J. Plant Breed.* 8 379–384. 10.5958/0975-928X.2017.00057.6

[B35] Harley R. M. Atkins S. Budantsev A. L. Cantino P. D. Conn B. J. Grayer R. (2004). “Labiatae,” in *Families and Genera of Vascular Plants*, Vol. 7 eds Kubitzki K. Kadereit J. W. (Berlin: Springer). 167–275.

[B36] Herrera-Arroyo M. L. Rico Y. Bedolla-García B. Y. (2020). Morphotype divergence and genetic diversity of Hedeoma piperita Benth. in western Mexico. *Mol. Biol. Rep.* 47 8925–8934. 10.1007/s11033-020-05946-x 33125598

[B37] Holland M. M. Parson W. (2011). GeneMarker^®^ HID: a reliable software tool for the analyses of forensic STR data. *J. Forensic Sci.* 56 29–35. 10.1111/j.1556-4029.2010.01565.x 20887353

[B38] Huang C. J. Chu F. H. Huang Y. S. Hung Y. M. Tseng Y. H. Pu C. E. (2020). Development and technical application of SSR-based individual identification system for *Chamaecyparis taiwanensis* against illegal logging convictions. *Sci. Rep.* 10:22095. 10.1038/s41598-020-79061-z 33328522 PMC7744516

[B39] Ihaka R. Gentleman R. (1996). R: a language for data analyses and graphics. *J. Comput. Graph. Stat.* 5 299–314. 10.1080/10618600.1996.10474713

[B40] Jarne P. Lagoda P. J. (1996). Microsatellites, from molecules to populations and back. *Trends Ecol. Evol.* 11 424–429. 10.1016/0169-5347(96)10049-5 21237902

[B41] Jiao S. Q. Dong A. X. Shi T. L. Liu H. Porth I. Xin H. B. (2020). Development of a large gene-associated SSR marker set and in-depth genetic characterization in scarlet sage. *Front. Genet.* 11:504. 10.3389/fgene.2020.00504 32508885 PMC7253628

[B42] Jin L. Macaubas C. Hallmayer J. Kimura A. Mignot E. (1996). Mutation rate varies among alleles at a microsatellite locus: phylogenetic evidence. *Proc. Natl. Acad. Sci. U.S.A.* 93 15285–15288. 10.1073/pnas.93.26.15285 8986803 PMC26396

[B43] Jose A. G. Concepción M. Francisco M. (2018). Trends in plant research using molecular markers. *Planta* 247 543–557. 10.1007/s00425-017-2829-y 29243155

[B44] Ju M. M. Fu Y. Zhao G. F. He C. Z. Li Z. H. Tian B. (2018). Effects of the Tanaka Line on the genetic structure of *Bombax ceiba* (Malvaceae) in dry-hot valley areas of southwest China. *Ecol. Evol.* 8 3599–3608. 10.1002/ece3.3888 29686841 PMC5901178

[B45] Kalia R. K. Chhajer S. Pathak R. (2020). Cross genera transferability of microsatellite markers from other members of family Bignoniaceae to *Tecomella undulata* (Sm.) Seem. *Acta Physiol. Plant.* 42:151. 10.1007/s11738-020-03138-5

[B46] Kalia R. K. Rai M. K. Kalia S. Singh R. Dhawan A. K. (2011). Microsatellite markers: an overview of the recent progress in plants. *Euphytica* 177 309–334. 10.1007/s10681-010-0286-9

[B47] Karaca M. Ince A. G. Aydin A. Ay S. T. (2013). Cross-genera transferable e-microsatellite markers for 12 genera of the *Lamiaceae* family. *J. Sci. Food Agric.* 93 1869–1879. 10.1002/jsfa.5982 23238626

[B48] Krak K. Vit P. Douda J. Mandak B. (2020). Development of 18 microsatellite markers for *Salvia pratensis*. *Appl. Plant Sci.* 8 e11316. 10.1002/aps3.11316 31993258 PMC6976894

[B49] Kwon S. J. Lee J. K. Kim N. S. Yu J. W. Dixit A. Cho E. G. (2005). Isolation and characterization of microsatellite markers in *Perilla frutescens* Brit. *Mol. Ecol. Notes* 5 455–457. 10.1111/j.1471-8286.2005.00901.x

[B50] Lepcha P. Sathyanarayana N. (2021). Variability for seed-based economic traits and genetic diversity analysis in Mucuna pruriens population of northeast India. *Agric. Res.* 1–11. 10.1007/s40003-021-00568-6

[B51] Lesser M. R. Parchman T. L. Buerkle C. A. (2012). Cross-species transferability of SSR loci developed from transciptome sequencing in lodgepole pine. *Mol. Ecol. Resour.* 12 448–455. 10.1111/j.1755-0998.2011.03102.x 22171820

[B52] Letunic I. Bork P. (2007). Interactive Tree of Life (iTOL): an online tool for phylogenetic tree display and annotation. *Bioinformatics* 23 127–128. 10.1093/bioinformatics/btl529 17050570

[B53] Leyva-Lopez N. Nair V. Bang W. Y. Cisneros-Zevallos L. Heredia J. B. (2016). Protective role of terpenes and polyphenols from three species of Oregano (*Lippia graveolens*, *Lippia palmeri* and *Hedeoma patens*) on the suppression of lipopolysaccharide-induced inflammation in RAW 264.7 macrophage cells. *J. Ethnopharmacol.* 187 302–312. 10.1016/j.jep.2016.04.051 27131433

[B54] Li C. H. Zheng Y. Q. Liu Y. Lin F. R. Huang P. (2021). Development of genomic SSR for the subtropical hardwood tree *Dalbergia* hupeana and assessment of their transferability to other related species. *Forests* 12 804–804. 10.3390/f12060804

[B55] Li K. Q. Chen L. Feng Y. H. Yao J. X. Li B. Xu M. (2014). High genetic diversity but limited gene flow among remnant and fragmented natural populations of *Liriodendron* chinense Sarg. *Biochem. Syst. Ecol.* 54 230–236. 10.1016/j.bse.2014.01.019

[B56] Li P. Qi Z. C. Liu L. X. Ohi-Toma T. Lee J. Hsieh T. H. (2017). Molecular phylogenetics and biogeography of the mint tribe Elsholtzieae (*Nepetoideae*, *Lamiaceae*), with an emphasis on its diversification in East Asia. *Sci. Rep.* 7:2057. 10.1038/s41598-017-02157-6 28515478 PMC5435694

[B57] Liang J. Y. Ning A. Q. Lu P. Y. An Y. Wang Z. L. Zhang J. (2021). Biological activities and synergistic effects of *Elsholtzia*stauntonii essential oil from flowers and leaves and their major constituents against *Tribolium castaneum*. *Eur. Food Res. Technol.* 247 2609–2619. 10.1007/s00217-021-03829-4

[B58] Lim S. E. Sa K. J. Lee J. K. (2021). Bulk segregant analysis identifies SSR markers associated with leaf and seed related traits in Perilla crop (*Perilla frutescens* L.). *Genes Genom.* 43 323–332. 10.1007/s13258-021-01056-5 33543373

[B59] Liu K. Muse S. V. (2005). PowerMarker: an integrated analyses environment for genetic marker analyses. *Bioinformatics* 21 2128–2129. 10.1093/bioinformatics/bti282 15705655

[B60] Liu Y. L. Geng Y. P. Song M. L. Zhang P. F. Hou J. L. Wang W. Q. (2019). Genetic structure and diversity of glycyrrhiza populations based on transcriptome SSR markers. *Plant Mole. Biol. Rep.* 37 401–412. 10.1007/s11105-019-01165-2

[B61] Lv J. H. Su X. H. Zhong J. J. (2012). Fumigant activity of *Elsholtzia stauntonii* extract against Lasioderma serricorne. *S. Afr. J. Sci.* 108 77–79. 10.4102/sajs.v108i7/8.556

[B62] Ma S. J. Sa K. J. Hong T. K. Lee J. K. (2017). Genetic diversity and population structure analysis in *Perilla frutescens* from Northern areas of China based on simple sequence repeats. *Genet. Mol. Res.* 16:16039746. 10.4238/gmr16039746 28973731

[B63] Mangaravite E. Vinson C. C. Rody H. V. S. Carniello M. A. Silva R. S. Oliveira L. O. (2016). Contemporary patterns of genetic diversity of *Cedrela fissilis* offer insight into the shaping of seasonal forests in eastern South America. *Am. J. Bot.* 103 307–316. 10.3732/ajb.1500370 26838366

[B64] Metzgar D. Bytof J. Wills C. (2000). Selection against frameshift mutations limits microsatellite expansion in coding DNA. *Genome Res.* 10 72–80. 10.1101/gr.10.1.72 10645952 PMC310501

[B65] Miranda K. M. C. Guimarães R. A. Silva M. J. Oliveira P. R. O. Ribeiro T. G. Mendes T. P. (2020). Cross-amplification and characterization of microsatellite markers in species of Manihot Mill. (*Euphorbiaceae*) endemic to the Brazilian Cerrado. *Acta Bot. Bras.* 34 772–777. 10.1590/0102-33062019abb0374

[B66] Moreira X. Abdala-Roberts L. Rasmann S. Castagneyrol B. Mooney K. A. (2016). Plant diversity effects on insect herbivores and their natural enemies: current thinking, recent findings, and future directions. *Curr. Opin. Insect Sci.* 14 1–7. 10.1016/j.cois.2015.10.003 27436639

[B67] Nadeem M. A. Nawaz M. A. Shahid M. Q. Dogan Y. Comertpay G. Yildiz M. (2018). DNA molecular markers in plant breeding: current status and recent advancements in genomic selection and genome editing. *Biotechnol. Biotechnol. Equip.* 32 261–285. 10.1080/13102818.2017.1400401

[B68] Nei M. Tajima F. Tateno Y. (1983). Accuracy of estimated phylogenetic trees from molecular data. *J. Mol. Evol.* 19 153–170. 10.1007/BF01840887 6571220

[B69] Ni Z. X. Bai T. D. Cai H. Chen S. F. Xu L. (2015). The Transferability of *Pinus massoniana* SSR in other *Pinus* species. *Mol. Plant Breed.* 13 2811–2817. 10.13271/j.mpb.013.002811

[B70] Oliveira E. J. Pádua J. G. Zucchi M. I. Vencovsky R. Vieira M. L. C. (2006). Origin, evolution and genome distribution of microsatellites. *Genet. Mol. Biol.* 29 294–307. 10.1590/S1415-47572006000200018

[B71] Pan L. Huang T. Yang Z. Tang L. Cheng Y. Wang J. (2018). EST-SSR marker characterization based on RNA-sequencing of Lolium multiflorum and cross transferability to related species. *Mol. Breed.* 38:80. 10.1007/s11032-018-0775-4

[B72] Park D. H. Sa K. J. Lim S. E. Ma S. J. Lee J. K. (2019). Genetic diversity and population structure of *Perilla frutescens* collected from Korea and China based on simple sequence repeats (SSRs). *Genes Genom.* 41 1329–1340. 10.1007/s13258-019-00860-4 31468347

[B73] Park S. D. E. (2001). *Trypanotolerance in West African Cattle and the Population Genetic Effects of Selection*. Dissertation’s thesis. Dublin: University of Dublin.

[B74] Peakall R. Gilmore S. Keys W. Morgante M. Rafalski A. (1998). Cross-species amplification of soybean (*Glycine max*) simple sequence repeats (SSRs) within the genus and other legume genera: implications for the transferability of SSRs in plants. *Mol. Biol. Evol.* 15 1275–1287. 10.1093/oxfordjournals.molbev.a025856 9787434

[B75] Peakall R. Smouse P. E. (2012). GenAlEx 6.5: genetic analysis in Excel. population genetic software for teaching and research—an update. *Bioinformatics* 28 2537–2539. 10.1093/bioinformatics/bts460 22820204 PMC3463245

[B76] Pebesma E. (2018). Simple features for R: standardized support for spatial vector data. *R J.* 10 439–446. 10.32614/RJ-2018-009

[B77] Pekhova O. A. Timasheva L. A. Danilova I. L. Belova I. V. (2020). Accumulation of biologically active substances in plants of *Elsholtzia*stauntonii Benth. grown in the foothill zone of the Crimea. *Agrar. Bullet. Urals.* 11 76–84. 10.32417/1997-4868-2020-202-11-76-84

[B78] Peng J. Y. Shi C. Wang D. W. Li S. Z. Zhao X. L. Duan A. N. (2021). Genetic diversity and population structure of the medicinal plant *Docynia delavayi* (Franch.) Schneid revealed by transcriptome-based SSR markers. *J. Appl. Res. Med. Aromat. Plants* 21:100294. 10.1016/j.jarmap.2021.100294

[B79] Pern Y. C. Lee S. Y. Ng W. L. Mohamed R. (2020). Cross-amplification of microsatellite markers across agarwood-producing species of the Aquilarieae tribe (*Thymelaeaceae*). *3 Biotech* 10:103. 10.1007/s13205-020-2072-2 32099744 PMC7007476

[B80] Pinto M. V. Poornima H. S. Sivaprasad V. Naik V. G. (2018). A new set of mulberry-specific SSR markers for application in cultivar identification and DUS testing. *J. Genet.* 97 e31–e37. 10.1007/s12041-018-0900-5 29700272

[B81] Pistelli L. Najar B. Giovanelli S. Lorenzini L. Tavarini S. Angelini L. G. (2017). Agronomic and phytochemical evaluation of lavandin and lavender cultivars cultivated in the Tyrrhenian area of Tuscany (Italy). *Ind. Crops Prod.* 109 37–44. 10.1016/j.indcrop.2017.07.041

[B82] Poljuha D. Franić M. Kralj I. Weber T. Šatović Z. Ban D. (2021). Genetic diversity and structure analysis of Croatian garlic collection assessed by SSR markers. *Folia Hortic.* 33 157–171. 10.2478/fhort-2021-0011

[B83] Powell W. Morgante M. Andre C. Hanafey M. Vogel J. Tingey S. (1996). The comparison of RFLP, RAPD, AFLP and SSR (microsatellite) markers for germplasm analysis. *Mol. Breed.* 2 225–238. 10.1016/j.scienta.2011.06.015

[B84] Pritchard J. K. Stephens M. Donnelly P. (2000). Inference of population structure using multilocus genotype data. *Genetics* 155 945–959. 10.1093/genetics/155.2.945 10835412 PMC1461096

[B85] Qi L. J. Long P. Jiang C. Yuan Y. Huang L. Q. (2015). Development of microsatellites and genetic diversity analysis of *Scutellaria baicalensis* Georgi using genomic-SSR markers. *Acta Pharm. Sin.* 50 500–505. 10.16438/j.0513-4870.2015.04.002 26223135

[B86] Radosavljević I. Jakse J. Javornik B. Satovic Z. Liber Z. (2011). New microsatellite markers for *Salvia officinalis* (*Lamiaceae*) and cross-amplification in closely related species. *Am. J. Bot.* 98 e316–e318. 10.3732/ajb.1000462 22003176

[B87] Radosavljević I. Satovic Z. Jakse J. Javornik B. Greguraš D. Jug-Dujaković M. (2012). Development of new microsatellite markers for *Salvia officinalis* L. and its potential use in conservation-genetic studies of narrow sndemic *Salvia* brachyodon Vandas. *Int. J. Mol. Sci.* 13 12082–12093. 10.3390/ijms130912082 23109901 PMC3472793

[B88] Radosavljević I. Satovic Z. Liber Z. (2015). Causes and consequences of contrasting genetic structure in sympatrically growing and closely related species. *AoB Plants* 7:106. 10.1093/aobpla/plv106 26333826 PMC4597123

[B89] Ren P. (2012). *The research on genetic diversity of Leonurus japonicus germplasm resources in Henan province. [master’s thesis].* Zhengzhou (HN): Henan Agricultural University.

[B90] Sa K. J. Choi I. Y. Park K. C. Lee J. K. (2018). Genetic diversity and population structure among accessions of *Perilla frutescens* (L.) Britton in East Asia using new developed microsatellite markers. *Genes Genom.* 40 1319–1329. 10.1007/s13258-018-0727-8 30105737

[B91] Sa K. J. Choi I. Y. Ueno M. Lee J. K. (2015). Genetic diversity and population structure in cultivated and weedy types of Perilla in East Asia and other countries as revealed by SSR marker. *Hortic. Environ. Biotechnol.* 56 524–534. 10.1007/s13580-015-0039-8

[B92] Sa K. J. Lim S. E. Choi I. Y. Park K. C. Lee J. K. (2019). Development and characterization of new microsatellite markers for *Perilla frutescens* (L.) Britton. *Am. J. Plant Sci.* 10 1623–1630. 10.4236/ajps.2019.109115

[B93] Saeed A. F. Wang R. Z. Wang S. H. (2016). Microsatellites in pursuit of microbial genome evolution. *Front. Microbiol.* 6:1462. 10.3389/fmicb.2015.01462 26779133 PMC4700210

[B94] Sandes S. S. Pinheiro J. B. Zucchi M. I. Monteiro M. Arrigoni-Blank M. F. Blank A. F. (2013). Development and characterization of microsatellite primers in *Pogostemon cablin* (*Lamiaceae*). *Genet. Mol. Res.* 12 2837–2840. 10.4238/2013.August.8.4 24065640

[B95] Satya P. Paswan P. K. Ghosh S. Majumdar S. Ali N. (2016). Confamiliar transferability of simple sequence repeat (SSR) markers from cotton (*Gossypium hirsutum* L.) and jute (*Corchorus olitorius* L.) to twenty two Malvaceous species. *3 Biotech* 6:65. 10.1007/s13205-016-0392-z 28330135 PMC4754293

[B96] Savić A. Pipan B. Vasić M. Meglič V. (2021). Genetic diversity of common bean (*Phaseolus vulgaris* L.) germplasm from Serbia, as revealed by single sequence repeats (SSR). *Sci. Hortic.* 288:110405. 10.1016/j.scienta.2021.110405

[B97] Schneider S. J. Hwang A. Y. Land S. D. Chen L. L. Thomas A. L. Hwang C. F. (2019). Genetic diversity of ten black walnut (*Juglans nigra* L.) cultivars and construction of a mapping population. *Tree Genet. Genomes* 15:62. 10.1007/s11295-019-1369-y

[B98] Segarra-Moragues J. G. Gleiser G. (2009). Isolation and characterisation of di and tri nucleotide microsatellite loci in *Rosmarinus officinalis* (*Lamiaceae*), using enriched genomic libraries. *Conserv. Genet.* 10 571–575. 10.1007/s10592-008-9572-7

[B99] Segarra-Moragues J. G. Marco Y. C. Castellanos M. C. Molina M. J. García-Fayos P. (2015). Ecological and historical determinants of population genetic structure and diversity in the Mediterranean shrub *Rosmarinus officinalis* (*Lamiaceae*). *Bot. J. Linn. Soc.* 180 50–63. 10.1111/boj.12353

[B100] Serrote C. M. L. Reiniger L. R. S. Silva K. B. Rabaiolli S. M. D. Stefanel C. M. (2020). Determining the polymorphism information content of a molecular marker. *Gene* 726:144175. 10.1016/j.gene.2019.144175 31726084

[B101] Shivakumar M. S. Ramesh S. Mohan Rao A. Udaykumar H. R. Keerthi C. M. (2017). Cross legume species/genera transferability of SSR markers and their utility in assessing transferability of SSR markers and their utility in assessing polymorphism among advanced breeding lines in Dolichos bean (*Lablab purpureus* L.). *Int. J. Curr. Microbiol. App. Sci.* 6 656–668. 10.20546/ijcmas.2017.608.083

[B102] Si S. X. Peng Z. F. (2017). Introduction and domestication and application of *Elsholtzia stauntonii* in the middle and lower reaches of the Yellow River Plain. *J. Anhui Agri. Sci.* 45:214. 10.13989/j.cnki.0517-6611.2017.04.053

[B103] Sulu G. Kacar Y. A. Polat I. Kitapci A. Turgutoglu E. Simsek O. (2020). Identification of genetic diversity among mutant lemon and mandarin varieties using different molecular markers. *Turk. J. Agric. For.* 44 465–478. 10.3906/tar-1909-67 31411186

[B104] Tadesse M. Kebede M. Girma D. (2021). Genetic diversity of Tef [*Eragrostis tef* (Zucc.) Trotter] as revealed by microsatellite markers. *Int. J. Genomics* 2021:6672397. 10.1155/2021/6672397 33977102 PMC8087483

[B105] Tian Y. L. Li J. Q. Wang W. H. Shi A. P. Wang H. L. (2007). The germplasm resource and ornamental character of the *Lamiaceae* in Beijing. *J. Beijing Univ. Agric.* 22 41–43. 10.13473/j.cnki.issn.1002-3186.2007.03.006

[B106] Vanavermaete D. Fostier J. Maenhout S. De Baets B. (2021). Deep scoping: a breeding strategy to preserve, reintroduce and exploit genetic variation. *Theor. Appl. Genet.* 134 3845–3861. 10.1007/s00122-021-03932-w 34387711 PMC8580937

[B107] Vieira M. L. C. Santini L. Diniz A. L. Munhoz C. D. F. (2016). Microsatellite markers: what they mean and why they are so useful. *Genet. Mol. Biol.* 39 312–328. 10.1590/1678-4685-GMB-2016-0027 27561112 PMC5004837

[B108] Vining K. J. Pandelova I. Hummer K. Bassil N. Contreras R. Neill K. (2019). Genetic diversity survey of *Mentha aquatica* L. and *Mentha suaveolens* Ehrh., mint crop ancestors. *Genet. Resour. Crop Evol.* 66 825–845. 10.1007/s10722-019-00750-4

[B109] Wang J. Shete S. (2012). Testing departure from Hardy-Weinberg proportions. *Methods Mol. Biol.* 850 77–102. 10.1007/978-1-61779-555-8_6 22307695

[B110] Wang S. Q. (2020). Genetic diversity and population structure of the endangered species *Paeonia* decomposita endemic to China and implications for its conservation. *BMC Plant Biol.* 20:510. 10.1186/s12870-020-02682-z 33167894 PMC7650209

[B111] Wang X. P. Wen H. Shang Z. W. Yang S. Xu J. Shen Q. (2017). Genetic relationship and genetic diversity of 13 *Perilla frutescens* varieties based on SSR markers. *Guizhou Agri. Sci.* 45 103–106.

[B112] Xing X. Q. Li M. D. Gong Y. M. Niu B. Hao N. Tian Y. L. (2019). Comparative analysis of volatile components in essential oil of *Elsholtzia*stauntonii and different organs. *South Chin. Agri.* 13 150–155. 10.19415/j.cnki.1673-890x.2019.02.077

[B113] Yang Y. Y. He R. Q. Zheng J. Hu Z. H. Wu J. Leng P. S. (2020). Development of EST-SSR markers and association mapping with floral traits in Syringa oblata. *BMC Plant Biol.* 20:436. 10.1186/s12870-020-02652-5 32957917 PMC7507607

[B114] Young A. Boyle T. Brown T. (1996). The population genetic consequences of habitat fragmentation for plants. *Trends Ecol. Evol.* 11 413–418. 10.1016/0169-5347(96)10045-8 21237900

[B115] Zeng Y. F. Zhang J. G. Abuduhamiti B. Wang W. T. Jia Z. Q. (2018). Phylogeographic patterns of the desert poplar in Northwest China shaped by both geology and climatic oscillations. *BMC Ecol. Evol.* 18:75. 10.1186/s12862-018-1194-1 29801429 PMC5970483

[B116] Zhang W. J. Yuan Q. J. Jiang D. Zhang Y. Q. Huang L. Q. (2014). Development and characterisation of microsatellite markers for the medicinal plant *Scutellaria baicalensis* (*Lamiaceae*). *Biochem. Syst. Ecol.* 54 267–271. 10.1016/j.bse.2014.01.005

[B117] Zhang Z. Y. Cui B. B. Mao J. F. Pang X. M. Li Y. Y. (2015). Novel polymorphic EST-derived microsatellite markers for the red-listed five needle pine, *Pinus* dabeshanensis. *Conserv. Genet. Resour.* 7 191–192. 10.1007/s12686-014-0329-y

[B118] Zhao Y. J. Gong X. (2015). Genetic divergence and phylogeographic history of two closely related species (*Leucomeris decora* and *Nouelia insignis*) across the’ Tanaka Line’ in southwest China. *BMC Evol. Biol.* 15:134. 10.1186/s12862-015-0374-5 26153437 PMC4495643

[B119] Zheng S. Kang S. Shen Y. Sun L. (1999). Three new c-methylated flavones from *Elsholtzia*stauntonii. *Planta Med.* 65 173–175. 10.1055/s-2006-960459 17260252

[B120] Zhou X. M. Li B. Y. Wang Y. J. (2014). Cultivation and management of *Elsholtzia*stauntonii. *Chin. Flowers Hortic.* 24 40–41.

[B121] Zhou X. M. Liu H. C. Li B. Y. (2016). Type and distribution of trichomes on the leaf epidermis of *Elsholtzia stautonii* and the secreting process of the glandular trichomes. *Acta Hortic. Sin.* 43 1555–1565. 10.16420/j.issn.0513-353x.2016-0154

[B122] Zigene Z. D. Asfaw B. T. Bitima T. D. (2021). Analysis of genetic diversity in rosemary (*Salvia* rosemarinus Schleid.) using SSR molecular marker for its management and sustainable use in Ethiopian genebank. *Genet. Resour. Crop Evol.* 68 279–293. 10.1007/s10722-020-00984-7

